# Reduced Graphene Oxide-Polydopamine-Gold Nanoparticles: A Ternary Nanocomposite-Based Electrochemical Genosensor for Rapid and Early *Mycobacterium tuberculosis* Detection

**DOI:** 10.3390/bios13030342

**Published:** 2023-03-04

**Authors:** Mansi Chaturvedi, Monika Patel, Neha Bisht, Shruti, Maumita Das Mukherjee, Archana Tiwari, D. P. Mondal, Avanish Kumar Srivastava, Neeraj Dwivedi, Chetna Dhand

**Affiliations:** 1 CSIR-Advanced Materials and Processes Research Institute, Hoshangabad Road, Bhopal 462026, India; 2 School of Biomolecular Engineering & Biotechnology UTD RGPV, Bhopal 462033, India; 3 Academy of Scientific and Innovative Research (AcSIR), Ghaziabad 201002, India; 4 Amity Institute of Applied Sciences, Amity University Uttar Pradesh, Delhi 201303, India

**Keywords:** electrochemical biosensor, *Mycobacterium tuberculosis*, polydopamine, reduced graphene oxide, gold nanoparticle

## Abstract

Tuberculosis (TB) has been a devastating human illness for thousands of years. According to the WHO, around 10.4 million new cases of tuberculosis are identified every year, with 1.8 million deaths. To reduce these statistics and the mortality rate, an early and accurate TB diagnosis is essential. This study offers a highly sensitive and selective electrochemical biosensor for *Mycobacterium tuberculosis* (*MTB*) detection based on a ternary nanocomposite of reduced graphene oxide, polydopamine, and gold nanoparticles (rGO-PDA-AuNP). Avidin-biotin coupling was used to bind the *MTB* probe DNA onto the rGO-PDA-AuNP modified glassy carbon electrode (ssDNA/avidin/rGO-PDA-AuNP). UV-Visible, Raman, XRD, and TEM were used to evaluate the structural and morphological characteristics of rGO-PDA-AuNP. Furthermore, DNA immobilization is validated using FESEM and FT-IR techniques. The modified electrodes were electrochemically analyzed using cyclic voltammetry (CV) and linear sweep voltammetry (LSV), and the results indicate that the produced electrode can detect target DNA up to 0.1 × 10^−7^ mM with 2.12 × 10^−3^ mA µM^−1^ sensitivity and a response time of 5 s. The constructed genosensor displayed high sensitivity and stability, and it also provides a unique strategy for diagnosing *MTB* at an early stage. Furthermore, our rGO-PDA-AuNP/GCE-based electrochemical platform has broad potential for creating biosensor systems for detecting various infectious pathogens and therapeutically significant biomarkers.

## 1. Introduction

Graphene is an ultrathin, 2D honeycomb-shaped sp^2^-bonded carbon atom sheet [1]. Graphene derivatives have emerged as flexible materials for a number of applications owing to their distinct properties and flexibility, such as high electrical conductivity, superior mechanical strength, robust electrocatalytic properties, and extended surface active sites [2]. Graphene’s outstanding properties make it a highly explored material for the development of a wide range of multifunctional devices, including optoelectronic devices, semiconductors, sensors, and biosensors [3]. However, due to very strong π–π stacking and *van der Waals* interactions, the sp^2^-rich graphene sheets tend to agglomerate irreversibly and thus pose difficulty in forming uniform dispersions/solutions [2,4]. Interestingly, reduced graphene oxide (rGO) is often utilized in place of pristine graphene due to its unique qualities, such as its higher surface area, electrochemical stability, and superior electrocatalytic stability [5]. As a result, it has the potential to improve electron transport and the electrochemical signal while simultaneously having a propensity to aggregate. However, the most often utilized reducing agents for directly reducing the rGO are hydrazine, hydroquinone, and NaBH_4_, all of which are very toxic and harmful.

Polydopamine presents a promising strategy for surface functionalization of GO while concurrently reducing it to rGO [6]. This method converts GO to rGO and coats the rGO surface with a nano-thick biocompatible PDA layer. Thus, using this bioinspired coating technique improves the processability of the rGO while enriching the rGO sheets with functional groups such as catechol, amine, and imine, which may be used to immobilize biomolecules covalently on the rGO surface with high density and significant alignment via a Schiff base and the Michael addition processes [7]. The PDA surface functionalization has also been shown to improve the electrocatalytic capabilities and charge transfer rate of GO by increasing its electroactive surface area, making this surface modification particularly important for electrochemical sensors and biosensor systems [6]. Another significant aspect of PDA lies in its chemical structure, which contains numerous functional groups like catechol, amine, and imine groups that can be used as a starting point for further covalent modifications or alterations [8]. Ruan et al. have developed an efficient amperometric biosensor with high detection sensitivity (28.4 μM mM^−1^cm^−2^), a low detection limit (0.1 μM), a short response time (<4 s), and a low Michaelis–Menten constant (6.77 mM) using the PDA/rGO nanocomposite system [8]. The authors observed a high surface-to-volume ratio, high enzyme absorption, and high conductivity of PDA/rGO, which enables direct electron transfer from the redox enzyme to the electrode surface via the PDA/rGO transducer matrix. Similarly, Palanisamy et al. demonstrated very sensitive and selective amperometric detection of chlorpromazine employing rGO/pDA as the electrode material [6].

Noble metal nanoparticles possess distinct physical and chemical attributes that make them excellent scaffolds for the fabrication of novel chemical and biological sensors. They are highly stable, have a high surface area, and exhibit catalytic properties, which exhibit advantages in improving the sensitivity, selectivity, stability, and detection potential of biosensors [9]. These versatile properties of AuNP aroused the interest of the researchers, and they have developed LSPR-based biosensors for the detection of various biomolecules, including creatinine [10], cardiac troponin I [11], alanine aminotransferase [12], acetylcholine [13], cholesterol [14], uric acid [15], and p-cresol [16]. Integration of the noble metal through metal chelation chemistry is seen to dramatically speed the electrochemical response of the sensing electrodes while boosting the sensitivity of the sensor system even in rGO/pDA-based sensing/biosensing systems. In this direction, Xu et al. [7] created an electrochemical sensing platform based on Au@PDA-rGO nanocomposite for the efficient detection of bisphenol A. The designed biosensor proved to be exceptionally sensitive, with a wide detection range and a very low detection limit. Furthermore, the biosensor may be utilized to detect BPA in plastic items. Huang et al. [17] also utilized an AgNPs-PDA@rGO nanocomposite to create an electrochemical DNA-based biosensor. The suggested biosensor demonstrated a strong selectivity for one-base mismatch DNA. Furthermore, Li et al. [18] used pDA-Lac-rGO-Pd nanocomposites to create a laccase-based biosensor with great stability, selectivity, and repeatability, as well as exceptional electrocatalytic capability toward phenolic contaminants. Additionally, Bueno et al. reported various impedimetric immunosensors for the detection of various biomarkers and pathogens [19].

Based on the aforementioned consideration, the present research work focuses on the development of a highly selective, rapid, and sensitive electrochemical biosensor for detecting oligonucleotide sequences specific to *Mycobacterium tuberculosis* (MTB). MTB is one of the most common causes of pulmonary tuberculosis, and in terms of yearly mortality toll, this disease has surpassed acquired immunodeficiency syndrome (AIDS), generating major health concerns. Additionally, people around the world are developing multi-drug-resistant tuberculosis (MDR-TB), and to drive the choice of antibiotics, a great research effort has been made to develop low-cost, sensitive, and fast biosensors. For instance, Brunetti et al. developed an optoelectronic biosensor for the monitoring of bacterial biofilm [20], and Wang et al. studied the response of the biofilms to different antibiotics by using confocal microscopy [21]. Additionally, Therisod et al. developed a label-free, nondestructive technique to distinguish Gram-bacteria [22], and for the investigation and monitoring of susceptibility at the single-bacterium level, Conteduca et al. developed an electro-photonic technique [23].

**Scheme 1:**
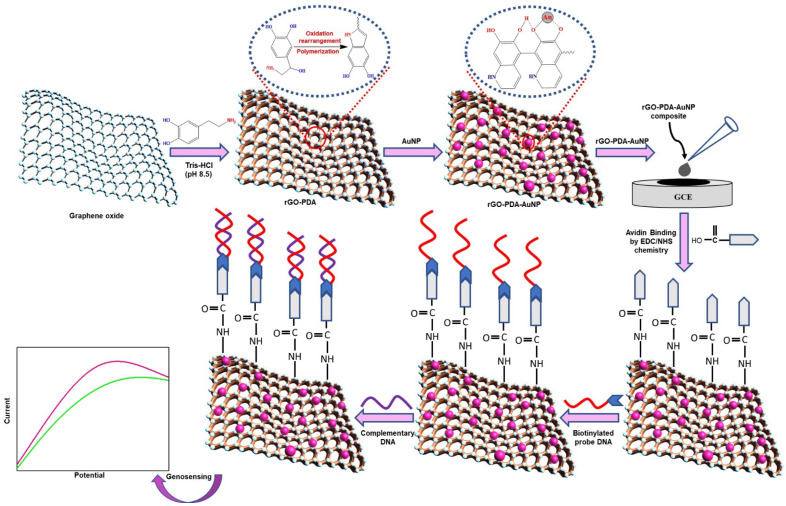
Showing the fabrication of a ssDNA/rGO-PDA-AuNP/GCE bioelectrode onto GCE.

In the present work, we aimed to develop a highly sensitive, selective, and stable electrochemical biosensor by immobilizing a probe containing DNA specific to MTB on the rGO-PDA-AuNP/GCE nanocomposite via an avidin-biotin coupling. The biosensing matrix was a nanocomposite of rGO, PDA, and AuNPs that was primarily integrated into the glassy carbon electrode (GCE) surface, providing a high electro-active surface area and enhanced electron transport for detection response. A detailed analysis of the rGO-PDA-AuNP nanocomposite and the ssDNA/rGO-PDA-AuNP/GCE bioelectrode with extensive biosensing experiments of the bioelectrode for MTB detection has been reported. Scheme 1 depicts the fabrication of a ssDNA/rGO-PDA-AuNP/GCE bioelectrode.

## 2. Materials and Methods

### 2.1. Chemicals

Graphene oxide utilized in the study has been prepared chemically using a modified Hummer’s method, as discussed in this section. Dopamine hydrochloride, gold nanoparticles (10 nm diameter, OD 1, stabilized in citrate buffer), N-hydroxy-succinimide (NHS), N-ethyl-N′-(3-dimethylaminopropyl carbodiimide) (EDC), avidin, potassium ferricyanide (III) [K_3_Fe(CN)_6_], potassium hexacyanoferrate trihydrate [K_4_Fe(CN)_6_·3H_2_O], sodium phosphate monobasic dihydrate (NaH_2_PO_4_·2H_2_O), sodium phosphate dibasic (Na_2_HPO_4_), were all purchased from Sigma-Aldrich. A biotinylated single-stranded probe DNA unique to *MTB*, complementary target DNA (cDNA), one-base mismatch DNA, and noncomplementary DNA were purchased from Sigma-Aldrich. The required reagents are prepared in deionized (DI) water, and all solutions and glassware have been autoclaved.

The oligonucleotide sequences used in this work are:

Biotinylated probe: biotin-5′-GGTCTTCGTGGCCGGCGTTCA-3′;

Complementary: 5′-TGAACGCCGGCCACGAAGACC-3′;

One-base mismatch: 5′-TGAACGCCGACCACGAAGACC-3′;

Noncomplementary: 5′-ATGTCTCAAGCCAGCTGCTG-3′.

### 2.2. Preparation of rGO-PDA/GCE Nanocomposite

Graphite flakes were used to synthesize GO via a modified Hummer’s method, as reported previously [24]. The self-polymerization approach of dopamine hydrochloride (DA) with concurrent reactions of GO was employed to prepare the rGO-PDA nanocomposite [25,26]. In this process, 50 mg of GO was first dissolved in 100 mL of 10 mM Tris HCL (pH 8.5), then 50 mg of DA was added and continuously stirred for 24 h at 60 °C. The color of the GO in the nanocomposite turned black from brown once the reaction was completed. Subsequently, the nanocomposite solution underwent centrifugation at 10,000× *g* RPM and was washed twice with deionized water. The solution was further lyophilized for 24 h to obtain rGO-PDA nanocomposite powder.

### 2.3. Preparation of rGO-PDA-AuNP/GCE Nanocomposite

About 2 mg mL^−1^ of the resulting rGO-PDA nanocomposite was re-dispersed in ethanol:water (2:1) followed by 60 min of sonication. In the meantime, different concentrations (10 µL, 50 µL, and 100 µL) of AuNPs were added to the rGO-PDA nanocomposite by keeping it in a magnetic stirrer for 30 min. Afterward, the selected concentration of AuNPs was used for electrochemical studies and sensing.

### 2.4. Electrode Modification

A GCE was utilized as a working electrode with a surface area of 5 mm for electrochemical studies. The GCE was cleaned with alumina slurry followed by 20 min of sonication. About 7 µL of rGO-PDA-AuNP nanocomposite was deposited onto the pre-cleaned surface of GCE and dried at room temperature or kept in a dry cabinet for 24 h.

### 2.5. Fabrication of Nuclei-Acid-Functionalized Modified GCE

By using Avidin-Biotin coupling, a single stranded biotinylated probe DNA (5 μL of 21-mer), unique to *MTB*, was covalently bound onto the avidin-modified rGO-PDA-AuNP/GCE surface. In addition, 15 mM EDC and 30 mM NHS were prepared using deionized water and utilized to activate a 10 μL avidin (1 mg/mL) solution followed by a 2 h incubation at 25 °C. The surface of rGO-PDA-AuNP/GCE was immobilized with 7 μL of activated avidin for 2 h. Additionally, the avidin-rGO-PDA-AuNP/GCE surface was immobilized with 5 µL of probe DNA unique to *MTB*, preceded by a 30 min incubation at 25 °C. Additionally, the fabricated ssDNA/rGO-PDA-AuNP/GCE bioelectrode was optimized for hybridization time and incubated for 5 min at 25 °C in the optimal concentration of the complementary target solution.

### 2.6. Instrumentation

The ultraviolet-visible spectra of nanocomposite materials were measured using an Evolution 220 UV-visible spectrophotometer from Thermo Fisher Scientific with DI as a reference in the range of 200–800 nm. Raman spectroscopy was done by using the Indi Ram CTR-300 Raman spectrometer, Technos Instruments, India, by using Diode Pump Solid State (DPSS) lasers with a wavelength of 532 nm, ranging from 1000 to 3500 cm^−1^. X-ray diffraction (XRD) patterns of the nanocomposites were examined using a d8 X-ray diffractometer using a wavelength of 1.54 Å and a monochromatic X-ray beam of Cu-Ka radiation. The functional group analysis of rGO-PDA-AuNP and ssDNA/rGO-PDA-AuNP was done by Fourier Transform Infrared Radiation (FT-IR), in the region of 400–4000 cm^−1^, using a Nicolet iS50 spectrometer. The elemental and morphological assessment of the nanocomposites was performed using field emission scanning electron microscopy (FESEM) and a high-resolution transmission electron microscope (HR-TEM). FESEM and Energy Dispersive X-ray Analysis (EDX) were performed using the Nova Nano SEM. Moreover, HR-TEM analysis was conducted at 200 keV using a JEOL JEM-F200 HR-TEM apparatus. The electrochemical investigation was conducted by using an Autolab Potentiostat/Galvanostat three-electrode system consisting of a platinum wire as a counter electrode (1 mm), a working electrode (GCE), and Ag/AgCl as a reference electrode. The CV and LSV were conducted in 0.1 M phosphate buffered saline (PBS) containing 10 Mm [Fe(CN)_6_]^3−/4−^ as a redox probe with a scan rate of 0.05 V/s.

## 3. Results and Discussion

### 3.1. Ultraviolet-Visible (UV-Vis) Spectroscopy

**Figure 1:**
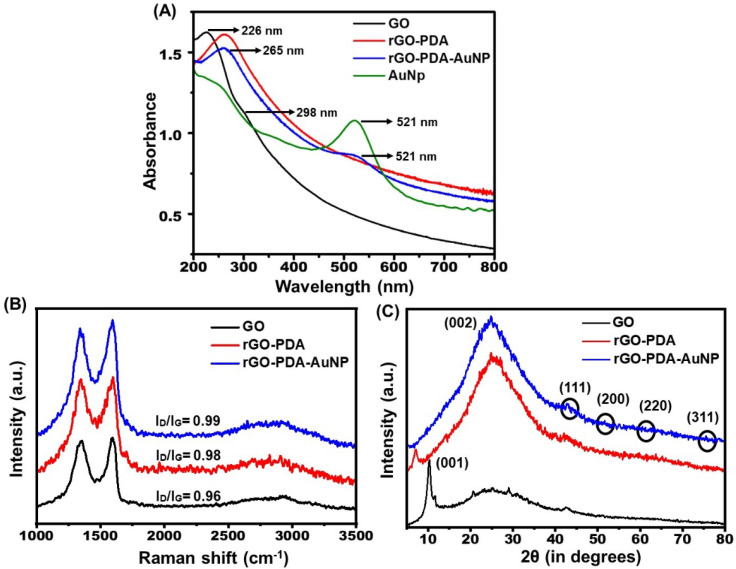
(**A**) UV-visible absorption spectra, (**B**) Raman spectra, and (**C**) X-ray diffraction patterns of GO, rGO-pDA, and rGO-pDA-AuNP composites.

UV-visible spectroscopy analysis was utilized to confirm the formation of GO, rGO-PDA, and rGO-PDA-AuNP ternary nanocomposites (Figure 1A). The GO spectra revealed a significant absorption peak at 226 nm as well as a shoulder peak at 298 nm. These peaks are associated with the π→π* transition of aromatic C=C bonds (alkenes and arenes) and the n→π* transition of the C=O bond [27]. The peak shifts from 226 to 265 nm following functionalization with PDA, showing that the bulk of GO has been reduced to rGO [28,29], and the change in color of the nanocomposite from brown to black further verifies the effective reduction of GO and polymerization of DA to pDA. Furthermore, the considerable increase in near-infrared (NIR) absorption confirms the conversion of DA to PDA [30]. These findings are compatible with the findings of Huang et al. [17] and Ruan et al. [8] indicating PDA functionalization, which resulted in the restoration of graphene’s electrical structure. The spectrum of AuNP show a prominent absorption peak at 521 nm, which is the characteristic peak of AuNP and is attributed to the surface plasmon resonance absorption peak of gold nanoparticles. Following the inclusion of AuNP on the rGO-PDA nanocomposite, an absorption peak at 521 nm was observed, suggesting that AuNP was effectively decorated on the surface of the rGO-PDA sheets [31].

### 3.2. Raman Spectroscopy

Raman spectroscopy was also used to investigate the as-prepared nanocomposite since it is a significant and more insightful approach for evaluating carbon materials. The spectra obtained by Raman spectroscopy for GO, rGO-PDA, and rGO-PDA-AuNP are shown in Figure 1B, indicating two distinctive peaks, the D band and the G band. The G band is assigned to the doubly degenerate phonon mode in the center of sp^2^ carbon networks, whereas the D band is attributed to defects in structure or disorder-induced phonon modes [6]. The D and G bands for GO are centered at 1354 and 1595 cm^−1^, but the band for rGO-PDA is identified at 1347 and 1597 cm^−1^. This minor change in the band is most likely caused by a reduction in GO [32]. The insertion of AuNPs on rGO-PDA nanocomposite sheets shifted the D and G bands to 1340 and 1594 cm^−1^, respectively. The D to G band intensity ratio (I_D_/I_G_) is associated with the level of the graphene disorder; hence, it is used to assess the degree of defects [33]. The I_D_/I_G_ increased from roughly 0.96 for GO to 0.98 for rGO-PDA owing to the restoration of the sp^2^ carbon skeleton, and an increase in edge defects is also observed as a result of PDA grafting on GO [34]. It indicates that rGO-PDA contains more segregated graphene domains than GO and that oxygen moieties are significantly reduced after PDA functionalization [35]. Furthermore, the increased intensity ratio reported for rGO-PDA-AuNP (0.99) correlates to an increase in interlayer distance [36] as well as the presence of AuNPs on the rGO-PDA nanocomposite [37].

### 3.3. X-ray Diffraction (XRD) Spectroscopy

To analyze the phase and crystalline structure of the produced nanocomposite, XRD measurements were taken, as shown in Figure 1C. The GO spectra revealed a strong and noticeable diffraction peak at 10.2°, which is related to the hexagonal crystalline structure of GO in the (001) plane [38]. This significant, unique peak of GO at 10.2° with an interplanar spacing of 8.66 is attributable to the insertion of oxygen moieties (carbonyl and epoxy groups) into the successive graphite layers due to the rigorous treatment of the chemical oxidation method used to produce GO [39]. The major peak of GO at 10.2° completely vanished upon PDA functionalization, and a wide peak appeared at 2θ = 24.9° with an interplanar distance of 3.58 Å demonstrating the effective reduction of GO by PDA and indicating the amorphous nature of PDA [34]. Furthermore, the integration of AuNPs into the rGO-PDA nanocomposite results in weak peaks related to AuNPs, which are attributed to the lower concentration of AuNPs. The diffraction peaks at 2θ of 42.7°, 51.6°, 63.2°, and 75°, respectively, are assigned to the (111), (200), (220), and (311) planes of the face-centered cubic (fcc) crystalline structure of Au, confirming the effective integration of AuNPs [40].

### 3.4. Transmission Electron Microscopy (TEM)

**Figure 2:**
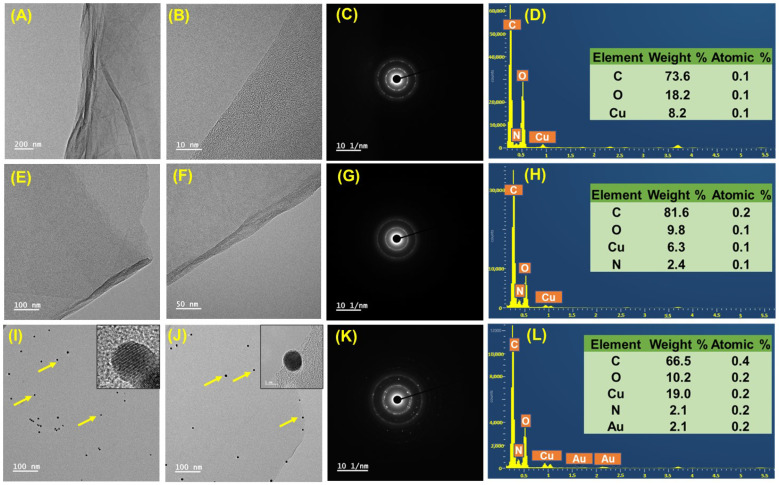
TEM micrograph of GO (**A**,**B**), rGO-PDA (**E**,**F**), AuNP (**I**), rGO-PDA-AuNP (**J**); SAED pattern of GO (**C**), rGO-PDA (**G**), rGO-PDA-AuNP (**K**), EDS of GO (**D**), rGO-PDA (**H**), and rGO-PDA-AuNP (**L**); yellow arrows indicate the sight of AuNPs in the nanocomposite in images (**I**,**J**). Inset images of (**I**,**J**) are HR-TEM images of AuNP in the respective sample.

TEM was used to examine the morphology and crystal structure of the material; Figure 2 shows the TEM micrographs of GO, rGO-PDA, and rGO-PDA-AuNP. The TEM pictures of GO show a transparent, layered, and wrinkled sheet-like structure, which are hallmarks of oxidized, exfoliated GO sheets (Figure 2A) [41]. The TEM image (Figure 2B) clearly shows the folding of GO over the borders, with lateral sizes ranging from a few nanometers to micrometers and an interlayer spacing of 0.35 nm validated by HR-TEM. The rGO-PDA sheets grow darker (Figure 2E) with increased contrast after being reduced and functionalized with PDA. When compared to GO, the rGO-PDA nanosheets revealed an outspread morphology (Figure 2F) and exhibited a rather rough structure, which was consistent with the reduction of functional groups (oxygen) while concurrently displaying the PDA layer’s covering on the GO’s surface [41]. The AuNP micrograph shown in Figure 2I reveals the homogenous dispersion of particles having a spherical shape and a uniform size of ~7.01 ± 0.92 nm. Interestingly, following the deposition of AuNPs on the surface of rGO-PDA, picture (Figure 2J) shows that spherical AuNPs are evenly distributed throughout the surface of rGO-PDA [30]. Upon deposition, the AuNP retains its spherical shape and size. The HR-TEM micrographs (inset Figure 2J) of rGO-PDA-AuNP reveal distinct lattice fringes, which indicate the high crystallinity of the material and further allow for the calculation of the interplanar spacing (d-spacing) using the Fast Fourier Transformation (FTT) algorithm in different areas of the HR-TEM images. The d-spacing found for rGO-PDA-AuNP is 2.42 Å, which corresponds to the crystal plane fcc (111). A TEM micrograph of a few-layer graphene sheet was also verified by selected area electron diffraction (SAED). Figure 2C depicts the non-overlapping graphene region indicating the multi-GO sheets, producing two clear hexagonal rings with discrete bright spots that are attributed to the polycrystalline nature of GO [36]. However, for rGO-PDA (Figure 2G) and rGO-PDA-AuNP (Figure 2K), similar diffraction spots with less intensity were found that could be ascribed to the non-uniformity of the graphene structure and the amorphous nature of PDA. In addition, the quantitative identification and elemental composition of GO, rGO-PDA, and rGO-PDA-AuNP were analyzed by EDS. The EDS spectrum of GO (Figure 2D) clearly reveals the presence of carbon and oxygen along with copper (ascribed to the copper grid that was used for TEM analysis). As for rGO-PDA (Figure 2H), there are several peaks in the EDS spectrum that correspond to the elements carbon, oxygen, nitrogen (attributed to the existence of an amine group in PDA), and copper. The lower oxygen content establishes the successful conversion of GO into rGO. In the rGO-PDA-AuNP elemental spectra (Figure 2L), the peak corresponding to gold is spotted in addition with carbon, oxygen, nitrogen, and copper, which demonstrates the successful incorporation of AuNPs on the rGO-PDA surface. The yellow arrow in Image I corresponds to the AuNP.

### 3.5. Field Emission Scanning Electron Microscopy (FESEM)

**Figure 3:**
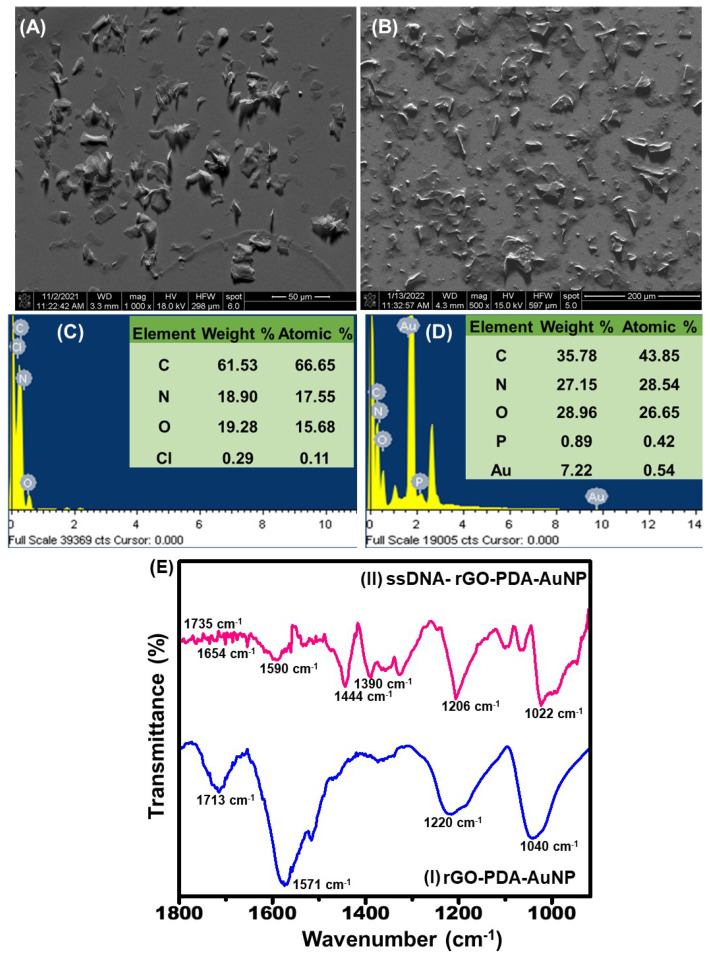
FESEM of (**A**) rGO-PDA, (**B**) ssDNA-rGO-PDA-AuNP, (**C**) EDX analysis of rGO-PDA and (**D**) ssDNA-rGO-PDA-AuNP, and (**E**) FTIR spectra of (I) rGO-PDA-AuNP and (II) ssDNA-rGO-PDA-AuNP.

To demonstrate the efficient immobilization of ssDNA on the rGO-PDA-AuNP, FESEM and EDX were used. The FESEM picture of the rGO-PDA composite (Figure 3A) displays a 3D morphology with rGO sheets covered in a uniformly thin layer of PDA. This might be attributed to the hydrophilic character of PDA, which is successfully coated onto RGO sheets [6]. Furthermore, FESEM images of ssDNA/rGO-PDA-AuNP (Figure 3B) show effective DNA binding, which results in smoothening and flattening of the rGO-PDA nanocomposite surface due to reduced porosity. This also suggests that the nanocomposite provides an advantageous environment for increased DNA probe loading [9]. The electron diffraction X-ray (EDX) analysis was used to obtain elemental information about rGO-PDA and the ssDNA/rGO-PDA-AuNP nanocomposite (Figure 3C,D). The EDX spectra of rGO-PDA demonstrate the availability of oxygen, carbon, and nitrogen in the nanocomposite. The presence of nitrogen, which is specific to the composition of PDA, demonstrates the homogeneous distribution of PDA over the surface of the rGO sheets. Whereas in the ssDNA/rGO-PDA-AuNP EDX spectra, the presence of phosphorus (ascribed to the presence of DNA) and gold (ascribed to the presence of gold nanoparticles) together with carbon, oxygen, and nitrogen suggests the effective synthesis of the nanocomposite and the immobilization of DNA onto the nanocomposite surface.

### 3.6. Fourier Transform Infrared Spectroscopy (FT-IR)

Furthermore, FTIR spectra were used to confirm the immobilization of the probe DNA on the rGO-PDA-AuNP surface. The IR spectra of rGO-PDA-AuNP (Figure 3E(I)) displays peaks at 1713, 1220, and 1040 cm^−1^, which are ascribed to C=O stretching of COOH groups, C-O stretching vibrations of epoxy groups, and C-O vibrations of alkoxy groups, respectively [42]. The 1571 cm^−1^ absorption peak is associated with the C=N stretching vibration, confirming the presence of PDA in the GO sheets. In the case of ssDNA/rGO-PDA-AuNP (Figure 3E(II)), the FTIR spectra shows many prominent peaks at 1735, 1590, and 1444 cm^−1^, which are caused by the C-O stretching of the purine and pyrimidine rings of DNA bases. Peaks at 1022, 1206, and 1390 cm^−1^ are ascribed to phosphate backbone P-O stretching. Furthermore, the signal seen at 1654 cm^−1^ is attributed to amide stretching vibrations, indicating the effective deposition of the MTB specific probe DNA onto the rGO-PDA-AuNP surface [9].

### 3.7. Electrochemical Response Studies

**Figure 4:**
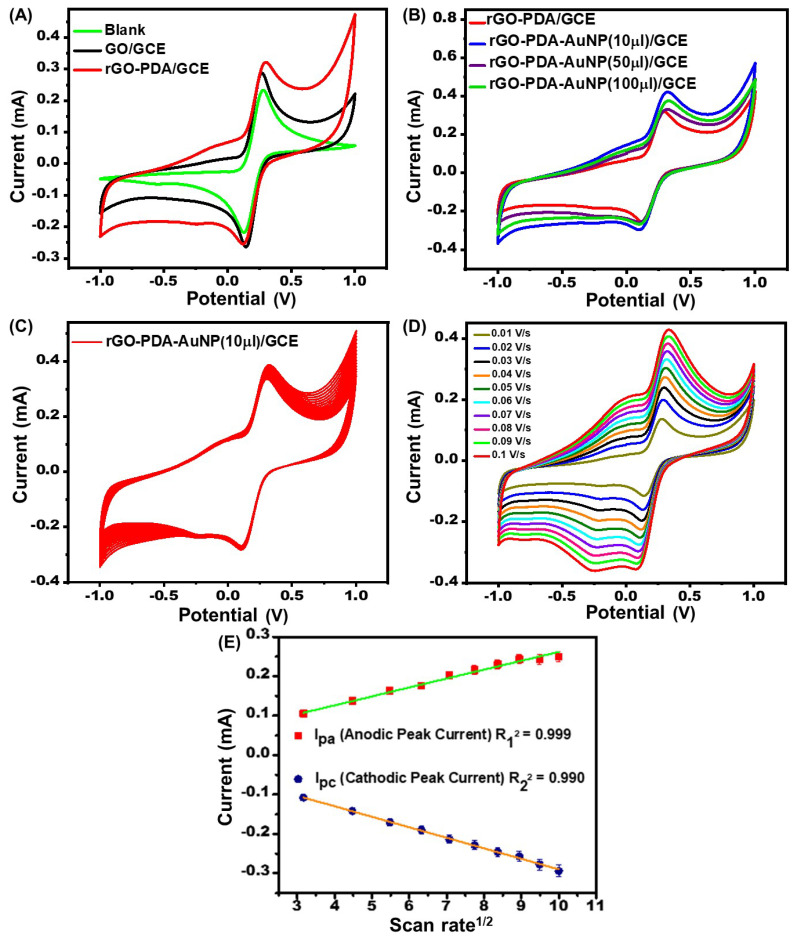
Cyclic voltammograms showing (**A**) comparison in electrochemical response of bare GCE, GO/GCE, and rGO-PDA/GCE; (**B**) modified electrode with 10 µL, 50 µL, and 100 µL AuNP; (**C**) 20 cycle stability curve of rGO-PDA-AuNP/GCE; (**D**) electrochemical response of rGO-PDA-AuNP/GCE at various scan rates; and (**E**) linear plot of current vs. square root of scan rate.

CV (Figure 4A) was used to study the electrochemical responses of bare GCE, GO/GCE, and rGO-PDA/GCE in the presence of 0.1 M PBS containing [Fe(CN)_6_]^3−/4−^ at pH 7.4, at a scanning rate of 0.05 Vs^−1^, and at potentials ranging from −1 V to +1 V. The cyclic voltammogram of GO/GCE displays a greater peak current (I_p_ = 0.29 mA) than bare GCE (I_p_ = 0.23 mA), indicating a larger surface-active area and considerably increased electrical conductivity, resulting in a rapid charge transfer rate [43]. The rGO-PDA/GCE had a higher redox peak current (I_pa_ = 0.32 mA) response and lower peak-to-peak potential separations (∆E_p_) than the bare GCE and GO/GCE. When compared to other electrodes, the rGO-PDA-AuNP/GCE had a greater redox peak current (I_pa_ = 0.42 mA) and a lower peak potential separation, showing that AuNPs had superior conductivity, a large specific surface area, and quick electron transit between the electrode surface and the solution [44]. Furthermore, the Randles–Sevcik Equation (1) is used to calculate the electroactive surface area (A_app_) value for rGO-PDA-AuNP/GCE, rGO-PDA/GCE, and GO/GCE [45].

(1) $$I_{p}=2.69\times 10^{5}n^{3/2}D^{1/2}v^{1/2}A_{app}C$$

where I_p_ is the peak current (A), D is the diffusion coefficient of [Fe(CN)_6_]^3−/4−^, n is the number of mobile electrons in [Fe(CN)_6_]^3−/4−^, ν is the scan rate (V/s^−1^), and C is the concentration of the [Fe(CN)_6_]^3−/4−^ solution (moles/cm^−3^). The A_app_ values for the rGO-PDA-AuNP/GCE, rGO-PDA/GCE, and GO/GCE are ~0.2715, ~0.2080, and ~0.1848 cm^2^.

Several concentrations of AuNPs ranging from 10 to 100 μL were utilized to establish the saturation threshold at which the current will reach its maximum value and stabilize. The modified GCE showed a greater current responsiveness of 0.42 mA after deposition with 10 μL of AuNP, as shown in Figure 4B. Furthermore, when 50 μL and 100 μL of AuNP are added, the modified GCE displays a lower current response of 0.33 mA and 0.37 mA, respectively, as compared to 10 μL of AuNP, indicating that the solution has reached the saturation point, after which the current begins to decline. Thus, the rGO-PDA-AuNP/GCE modified electrode with a 10 μL concentration of AuNPs had the highest electrocatalytic property and peak current and was chosen as the final nanocomposite material for biosensor development and is referred to as the rGO-PDA-AuNP nanocomposite throughout the rest of the manuscript. The electrochemical stability of the fabricated rGO-PDA-AuNP/GCE electrode was tested for up to 20 cycles at a scan rate of 0.05 Vs^−1^ between −0.1 and 0.1 V. The overlapping cyclic voltammograms (Figure 4C) showed that the rGO-PDA-AuNP modified electrode has good electrochemical stability. Although we only utilized one size of AuNP to optimize the electrochemical response in this study, it is noteworthy that there are publications available where the authors have thoroughly examined the influence of different sizes and shapes of AuNPs on the electrochemical and optical responses of the sensing platform and discussed their effect on the biosensing properties. Bonanni et al., [46] and Hu et al., [47] have done excellent research showing the impact of size and shape of AuNP on electrochemical response.

### 3.8. Impact of Scan Rate

The impact of scan rate on the electrochemical behavior of rGO-PDA-AuNP/GCE was examined by cyclic voltammetry to look into the correlation between the scan rate and the current of reduction and oxidation peaks. Figure 4D depicts the CV responsiveness of rGO-PDA-AuNP/GCE at different scan rates. By making an increment in the scan rate, the increase in Ep values induced by a positive shift in anodic peak current (I_pa_) and a negative shift in cathodic peak current (I_pc_) confirms the quasi-reversible process occurring at the electrode’s surface [41]. Furthermore, Figure 4E shows that the redox peak current of rGO-PDA-AuNP/GCE displayed a linear connection with the square root of scanning speeds ranging from 0.01 to 0.1 Vs^−1^, with R^2^ values of 0.93587 for I_pa_ and 0.96045 for I_pc_ with a 5% error.

### 3.9. Electrochemical Sensing

**Figure 5:**
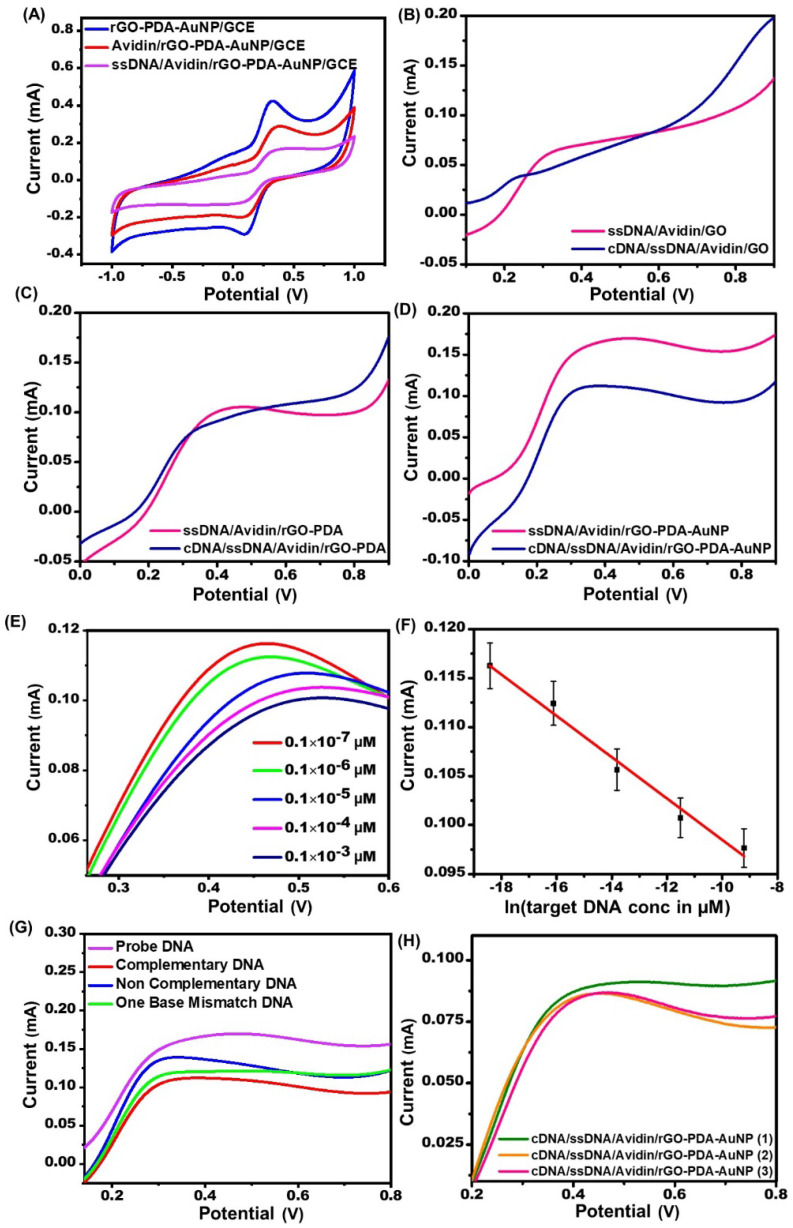
(**A**) CV comparing the electrochemical responses of rGO-PDA-AuNP/GCE, avidin-rGO-PDA-AuNP/GCE, and ssDNA-avidin-rGO-PDA-AuNP/GCE electrodes, sensing response of (**B**) ssDNA-avidin-GO, (**C**) ssDNA-avidin-rGO-PDA, and (**D**) ssDNA-avidin-rGO-PDA-AuNP bioelectrodes for CDNA specific to MTB. (**E**) LSV showing variation in the electrochemical response of the ssDNA/avidin/rGO-PDA-AuNP/GCE bioelectrode after hybridization with various concentrations of CDNA from 0.1 × 10^−7^ to 0.1 × 10^−3^ µM. (**F**) A linear plot of I_p_ versus ln complementary/target DNA. (**G**) LSV showing the variation in LSV responsiveness of the ssDNA/avidin/rGO-PDA-AuNP/GCE bioelectrode upon hybridizing with CDNA, noncomplementary DNA, and a one-base mismatch DNA sequence. (**H**) LSV plots revealing the reproducibility of the ssDNA/avidin/rGO-PDA-AuNP/GCE bioelectrodes.

The electrochemical experiments on rGO-PDA-AuNP/GCE, avidin/rGO-PDA-AuNP/GCE, and ssDNA/avidin/rGO-PDA-AuNP/GCE electrodes (Figure 5A) show distinct redox peaks at 0.32, 0.36, and 0.47 V, with a significant reduction in I_p_ from 0.43 to 0.17 mA following DNA immobilization. The decrease in current might be attributed to repulsion between negatively charged DNA deposited on the rGO-PDA-AuNP/GCE and [Fe(CN)_6_]^3−/4−^ redox probes in the PBS, resulting in slower electron transport [9]. The electrochemical sensing responses of mono (ssDNA/avidin/GO), bi (ssDNA/avidin/rGO-PDA), and tri-component (ssDNA/avidin/rGO-PDA-AuNP)-based bioelectrodes were compared in Figure 5B–D which shows that the tri-component nanocomposite has a better electrochemical sensing response (ΔI = 0.05 mA) as compared to mono (ΔI = 0.01 mA) and bi-component (ΔI = 0.01 mA) upon incubating with a specific concentration (0.1 × 10^−3^ µM) of complementary target DNA. Furthermore, the electrochemical response was recorded by incubating the bioelectrodes with varying CDNA concentrations for 5 mins at 25 °C. The LSV results of ssDNA/avidin/rGO-PDA-AuNP/GCE with varied concentrations ranging from 0.1 × 10^−3^ to 0.1 × 10^−7^ µM of MTB-specific CDNA are depicted in Figure 5E. The oxidation current increases linearly with increasing CDNA concentrations, from 0.1 × 10^−3^ to 0.1 × 10^−7^ µM. Furthermore, the maximum drop in oxidation current is observed at 0.1 × 10^−3^ µM, showing that this concentration is sufficient to saturate the surface of the ssDNA/avidin/rGO-PDA-AuNP/GCE bioelectrode. According to the findings, the ssDNA/avidin/rGO-PDA-AuNP/GCE bioelectrode has a very low detection limit of 0.1 × 10^−7^ µM. The quantitative decrease in the ssDNA/avidin/rGO-PDA-AuNP/GCE oxidation I_p_ as a measure of CDNA concentration is shown in Figure 5F. Based on the slope of the linear regression curve (Equation (2)), the sensitivity of the ssDNA/avidin/rGO-PDA-AuNP/GCE bioelectrode was calculated to be 2.12 × 10^−3^ mA µM^−1^ with a 2% error.

(2) $$I\left(mA\right)=2.12\times 10^{-3}[\ln(\mathrm{target}\;\mathrm{DNA}\;\mathrm{conc}.\;\mathrm{in}\;\mu M)]+7.71\times 10^{-2},R^{2}=0.98$$

To check the selectivity of the biosensor, the ssDNA-avidin-rGO-PDA-AuNP/GCE bioelectrode was incubated with an equal concentration (0.1 × 10^−3^ µM) of CDNA, one-base mismatch DNA, and noncomplementary DNA, and their LSV responses were recorded after 15 min as depicted in Figure 5G. Expectedly, CDNA incubation causes the greatest decline in peak current, demonstrating successful CDNA hybridization with the probe DNA immobilized on the bioelectrode surface. However, when incubated with one-base mismatch DNA under comparable circumstances, a small change in peak current was detected. Furthermore, when treated with noncomplementary DNA, the peak current was not significantly changed, indicating the selectivity of bioelectrodes for detecting MTB-specific CDNA. Additionally, by modifying three different ssDNA-avidin-rGO-PDA-AuNP/GCE bioelectrodes with 0.1 × 10^−3^ µM CDNA, the repeatability of the biosensor was tested (Figure 5H). All the modified electrodes showed consistent results with only 0.012% relative standard deviation. Table 1 shows the comparison of the ssDNA/rGO-PDA-AuNP/GCE bioelectrode with other carbon nanomaterials.

## 4. Conclusions

Nanocomposites are receiving a lot of attention in the current state of advanced nanoscience because they open up a new horizon for unique capabilities in clinical and non-clinical diagnostics. We developed and utilized a bioelectrode based on a rGO-PDA-AuNP/GCE nanocomposite as an extremely sensitive and effective platform for detecting DNA unique to *Mycobacterium tuberculosis*. Morphological investigations clearly show the integration of ssDNA, resulting in a smoother and flatter nanocomposite surface. Furthermore, we demonstrated the binding of ssDNA with the targeted complementary DNA onto the rGO-PDA-AuNP modified electrode surface. The ssDNA/rGO-PDA-AuNP/GCE bioelectrode has been shown to be very stable, sensitive, and effective in detecting targeted MTB-specific CDNA with a detection limit of 0.1 × 10^−7^ µM, and a sensitivity of 2.12 × 10^−3^ mA µM^−1^, with a response time of 5 s. This fascinating platform offers an accessible, versatile, and effective tool that should be utilized and researched further for the development of enhanced electrochemical biosensors for other clinically relevant illnesses such as dengue, COVID-19, Alzheimer’s disease, cancer, and others.

## Figures and Tables

**Table 1:** Comparison of the biosensing properties of the ssDNA/rGO-PDA-AuNP/GCE bioelectrode with other carbon nanomaterials-based genoelectrodes for *MTB* detection.

Matrix Used	Method of Immobilization	Detection Limit	Linear Range of Detection	Response Time	Reference
Probe/ZrO_2_-CHIT/ITO	Covalent immobilization	7.8 × 10^−10^ M	From 7.8 × 10^−10^ to 5 × 10^–8^ M	1 min	[9]
rGO/Au/PANI nanocomposite	Covalent binding	1.0 × 10^−15^ M	From 1.0 × 10^−15^ to 1.0 × 10^−9^ M	-	[48]
PANI/GP composite nanofibers	Covalent binding via Avidin-Biotin coupling	7.853 × 10^−7^ M	From 10^−6^ to 10^−9^ M	30 min	[49]
AuNTsA (gold nanotube array)	Covalent binding	1.0 × 10^−12^ M	From 1.0 × 10^−12^ to 1.46 × 10^−8^ M	-	[50]
GF/ITO	Covalent binding	4.2 × 10^−13^ M	From 4.2 × 10^−13^ M to 4.2 × 10^−5^ M	-	[51]
Au/RGONR	Covalent binding	1 × 10^−16^ M	From 1 × 10^−16^ to 10^−6^ M	-	[52]
rGO-PDA-AuNP/GCEs	Covalent binding via Avidin-Biotin coupling	1.0 × 10^−14^ M	From 1.0 × 10^−14^ M to 1.0 × 10^−10^ M	5 s	Present work

Abbreviations used: ZrO_2_—Zirconium oxide, CHIT—Chitosan, ITO—Indium−tin-oxide, PANI—Polyaniline, GP—Graphene, GF—Graphene flakes, and RGONR—reduced graphene oxide nanoribbons.

## Data Availability

We would like to provide the data on request.

## References

[1] Yin Z., Sun S., Salim T., Wu S., Huang X., He Q., Lam Y.M., Zhang H. (2010). Organic photovoltaic devices using highly flexible reduced graphene oxide films as transparent electrodes. ACS Nano.

[2] Smith A.T., LaChance A.M., Zeng S., Liu B., Sun L. (2019). Synthesis, properties, and applications of graphene oxide/reduced graphene oxide and their nanocomposites. Nano Mater. Sci..

[3] Ghosh S., Sarker B.K., Chunder A., Zhai L., Khondaker S.I. (2010). Position dependent photodetector from large area reduced graphene oxide thin films. Appl. Phys. Lett..

[4] Xu Y., Bai H., Lu G., Li C., Shi G. (2008). Flexible graphene films via the filtration of water-soluble noncovalent functionalized graphene sheets. J. Am. Chem. Soc..

[5] Qiu X., Kundu C.K., Li Z., Li X., Zhang Z. (2019). Layer-by-layer-assembled flame-retardant coatings from polydopamine-induced in situ functionalized and reduced graphene oxide. J. Mater. Sci..

[6] Palanisamy S., Thirumalraj B., Chen S.-M., Wang Y.-T., Velusamy V., Ramaraj S.K. (2016). A Facile Electrochemical Preparation of Reduced Graphene Oxide@Polydopamine Composite: A Novel Electrochemical Sensing Platform for Amperometric Detection of Chlorpromazine. Sci. Rep..

[7] Xu X., Zheng Q., Bai G., Song L., Yao Y., Cao X., Liu S., Yao C. (2017). Polydopamine induced in-situ growth of Au nanoparticles on reduced graphene oxide as an efficient biosensing platform for ultrasensitive detection of bisphenol A. Electrochim. Acta.

[8] Ruan C., Shi W., Jiang H., Sun Y., Liu X., Zhang X., Sun Z., Dai L., Ge D. (2013). One-pot preparation of glucose biosensor based on polydopamine–graphene composite film modified enzyme electrode. Sens. Actuators B Chem..

[9] Das M., Dhand C., Sumana G., Srivastava A., Nagarajan R., Nain L., Iwamoto M., Manaka T., Malhotra B. (2011). Electrophoretic fabrication of chitosan− zirconium-oxide nanobiocomposite platform for nucleic acid detection. Biomacromolecules.

[10] Du H., Chen R., Du J., Fan J., Peng X. (2016). Gold nanoparticle-based colorimetric recognition of creatinine with good selectivity and sensitivity. Ind. Eng. Chem. Res..

[11] Wang Y., Singh R., Li M., Min R., Wu Q., Kaushik B.K., Jha R., Zhang B., Kumar S. (2022). Cardiac Troponin I Detection using Gold/Cerium-Oxide Nanoparticles assisted Hetro-Core Fiber Structure. IEEE Trans. NanoBiosci..

[12] Feng J., Gao J., Yang W., Liu R., Shafi M., Zha Z., Liu C., Xu S., Ning T., Jiang S. (2022). LSPR optical fiber sensor based on 3D gold nanoparticles with monolayer graphene as a spacer. Opt. Express.

[13] Zhu G., Wang Y., Wang Z., Singh R., Marques C., Wu Q., Kaushik B.K., Jha R., Zhang B., Kumar S. (2021). Localized plasmon-based multicore fiber biosensor for acetylcholine detection. IEEE Trans. Instrum. Meas..

[14] Kumar S., Kaushik B.K., Singh R., Chen N.-K., Yang Q.S., Zhang X., Wang W., Zhang B. (2019). LSPR-based cholesterol biosensor using a tapered optical fiber structure. Biomed. Opt. Express.

[15] Singh L., Singh R., Zhang B., Cheng S., Kaushik B.K., Kumar S. (2019). LSPR based uric acid sensor using graphene oxide and gold nanoparticles functionalized tapered fiber. Opt. Fiber Technol..

[16] Zhou Z., Wada K., Tong L. (2021). Nanophotonics and Micro/Nano Optics VII.

[17] Huang K.-J., Liu Y.-J., Wang H.-B., Wang Y.-Y. (2014). A sensitive electrochemical DNA biosensor based on silver nanoparticles-polydopamine@ graphene composite. Electrochim. Acta.

[18] Li D.-W., Luo L., Lv P.-F., Wang Q.-Q., Lu K.-Y., Wei A.-F., Wei Q.-F. (2016). Synthesis of polydopamine functionalized reduced graphene oxide-palladium nanocomposite for laccase based biosensor. Bioinorg. Chem. Appl..

[19] Leva-Bueno J., Peyman S.A., Millner P. (2020). A review on impedimetric immunosensors for pathogen and biomarker detection. Med. Microbiol. Immunol..

[20] Brunetti G., Conteduca D., Armenise M.N., Ciminelli C. (2021). Novel micro-nano optoelectronic biosensor for label-free real-time biofilm monitoring. Biosensors.

[21] Wang Y., Reardon C.P., Read N., Thorpe S., Evans A., Todd N., Van Der Woude M., Krauss T.F. (2020). Attachment and antibiotic response of early-stage biofilms studied using resonant hyperspectral imaging. NPJ Biofilms Microbiomes.

[22] Therisod R., Tardif M., Marcoux P.R., Picard E., Jager J.-B., Hadji E., Peyrade D., Houdré R. (2018). Gram-type differentiation of bacteria with 2D hollow photonic crystal cavities. Appl. Phys. Lett..

[23] Conteduca D., Brunetti G., Dell’Olio F., Armenise M.N., Krauss T.F., Ciminelli C. (2019). Monitoring of individual bacteria using electro-photonic traps. Biomed. Opt. Express.

[24] Kumar P., Shahzad F., Yu S., Hong S.M., Kim Y.-H., Koo C.M. (2015). Large-area reduced graphene oxide thin film with excellent thermal conductivity and electromagnetic interference shielding effectiveness. Carbon.

[25] Patel M., Bisht N., Prabhakar P., Sen R.K., Kumar P., Dwivedi N., Ashiq M., Mondal D., Srivastava A.K., Dhand C. (2023). Ternary nanocomposite-based smart sensor: Reduced graphene oxide/polydopamine/alanine nanocomposite for simultaneous electrochemical detection of Cd2+, Pb2+, Fe2+, and Cu2+ ions. Environ. Res..

[26] Huang N., Zhang S., Yang L., Liu M., Li H., Zhang Y., Yao S. (2015). Multifunctional electrochemical platforms based on the Michael addition/Schiff base reaction of polydopamine modified reduced graphene oxide: Construction and application. ACS Appl. Mater. Interfaces.

[27] Wang Z., Gou G., Shi L., Yang J., Xu C., Zhang L., Fan A., Min Y. (2018). Gold nanoparticle ensemble on polydopamine/graphene nanohybrid as a novel electrocatalyst for determination of baicalein. J. Appl. Polym. Sci..

[28] Hessein A., El-Moneim A. (2015). Developing Cost Effective Graphene Conductive Coating and its Application as Counter Electrode for CdS Quantum Dot Sensitized Solar Cell. Proc. World Congr. New Technol. (NewTech 2015).

[29] Li Y., Li Y., Yu X., Sun Y. (2019). Electrochemical determination of carbofuran in tomatoes by a concanavalin A (con A) polydopamine (PDA)-reduced graphene oxide (RGO)-gold nanoparticle (GNP) glassy carbon electrode (GCE) with immobilized acetylcholinesterase (AChE). Anal. Lett..

[30] Yuan Y.-G., Gurunathan S. (2017). Combination of graphene oxide–silver nanoparticle nanocomposites and cisplatin enhances apoptosis and autophagy in human cervical cancer cells. Int. J. Nanomed..

[31] Weng C.-H., Huang C.-C., Yeh C.-S., Lei H.-Y., Lee G.-B. (2008). Synthesis of hexagonal gold nanoparticles using a microfluidic reaction system. J. Micromech. Microeng..

[32] Silva C., Simon F., Friedel P., Pötschke P., Zimmerer C. (2019). Elucidating the chemistry behind the reduction of graphene oxide using a green approach with polydopamine. Nanomaterials.

[33] Wang S., Zhu J., Rao Y., Li B., Zhao S., Bai H., Cui J. (2019). Polydopamine modified graphene oxide-TiO2 nanofiller for reinforcing physical properties and anticorrosion performance of waterborne epoxy coatings. Appl. Sci..

[34] Ha Y.-M., Kim Y.N., Jung Y.C. (2021). Rapid and local self-healing ability of polyurethane nanocomposites using photothermal polydopamine-coated graphene oxide triggered by near-infrared laser. Polymers.

[35] Hidayah N., Liu W.-W., Lai C.-W., Noriman N., Khe C.-S., Hashim U., Lee H.C. (2017). Comparison on graphite, graphene oxide and reduced graphene oxide: Synthesis and characterization. AIP Conference Proceedings.

[36] Faiz M.A., Azurahanim C.C., Raba’ah S.A., Ruzniza M.Z. (2020). Low cost and green approach in the reduction of graphene oxide (GO) using palm oil leaves extract for potential in industrial applications. Results Phys..

[37] Palanisamy S., Thangavelu K., Chen S.-M., Thirumalraj B., Liu X.-H. (2016). Preparation and characterization of gold nanoparticles decorated on graphene oxide@ polydopamine composite: Application for sensitive and low potential detection of catechol. Sens. Actuators B Chem..

[38] Banumathi S., Uma J., Ravi A., Balraj B., Siva C., Ilanchezhiyan P., Kumar G.M. (2021). Rapid sun-light driven photocatalytic functions of 3D rGO/ZnO/Ag heterostructures via improved charge transfer kinetics. J. Mater. Res. Technol..

[39] Ci L., Song L., Jin C., Jariwala D., Wu D., Li Y., Srivastava A., Wang Z., Storr K., Balicas L. (2010). Atomic layers of hybridized boron nitride and graphene domains. Nat. Mater..

[40] Krishnamurthy S., Esterle A., Sharma N.C., Sahi S.V. (2014). Yucca-derived synthesis of gold nanomaterial and their catalytic potential. Nanoscale Res. Lett..

[41] Yan Q., Zhi N., Yang L., Xu G., Feng Q., Zhang Q., Sun S. (2020). A highly sensitive uric acid electrochemical biosensor based on a nano-cube cuprous oxide/ferrocene/uricase modified glassy carbon electrode. Sci. Rep..

[42] Jing Y., Yuan X., Yuan Q., He K., Liu Y., Lu P., Li H., Li B., Zhan H., Li G. (2016). Determination of nicotine in tobacco products based on mussel-inspired reduced graphene oxide-supported gold nanoparticles. Sci. Rep..

[43] Fu L., Lai G., Yu A. Electroanalysis of Dopamine Using Polydopamine Functionalized Reduced Graphene Oxide-Gold Nanocomposite. Proceedings of the 1st International Electronic Conference on Materials.

[44] Shi L., Wang Z., Chen X., Yang G., Liu W. (2019). Reduced Graphene Oxide/Polydopamine/Gold Electrode as Elecrochemical Sensor for Simultaneous Determination of Ascorbic Acid, Dopamine, and Uric Acid. Int. J. Electrochem. Sci.

[45] Duoc P.N.D., Binh N.H., Van Hau T., Thanh C.T., Van Trinh P., Tuyen N.V., Van Quynh N., Van Tu N., Chinh V.D., Thu V.T. (2020). A novel electrochemical sensor based on double-walled carbon nanotubes and graphene hybrid thin film for arsenic (V) detection. J. Hazard. Mater..

[46] Bonanni A., Pumera M., Miyahara Y. (2011). Influence of gold nanoparticle size (2–50 nm) upon its electrochemical behavior: An electrochemical impedance spectroscopic and voltammetric study. Phys. Chem. Chem. Phys..

[47] Hu J., Wang Z., Li J. (2007). Gold nanoparticles with special shapes: Controlled synthesis, surface-enhanced Raman scattering, and the application in biodetection. Sensors.

[48] Liu C., Jiang D., Xiang G., Liu L., Liu F., Pu X. (2014). An electrochemical DNA biosensor for the detection of Mycobacterium tuberculosis, based on signal amplification of graphene and a gold nanoparticle–polyaniline nanocomposite. Analyst.

[49] Mohamad F.S., Mat Zaid M.H., Abdullah J., Zawawi R.M., Lim H.N., Sulaiman Y., Abdul Rahman N. (2017). Synthesis and characterization of polyaniline/graphene composite nanofiber and its application as an electrochemical DNA biosensor for the detection of Mycobacterium tuberculosis. Sensors.

[50] Torati S.R., Reddy V., Yoon S.S., Kim C. (2016). Electrochemical biosensor for Mycobacterium tuberculosis DNA detection based on gold nanotubes array electrode platform. Biosens. Bioelectron..

[51] Mukherjee M.D., Dhand C., Dwivedi N., Singh B.P., Sumana G., Agarwal V.V., Tawale J.S., Malhotra B.D. (2015). Facile synthesis of 2-dimensional transparent graphene flakes for nucleic acid detection. Sens. Actuators B Chem..

[52] Mogha N.K., Sahu V., Sharma R.K., Masram D.T. (2018). Reduced graphene oxide nanoribbon immobilized gold nanoparticle based electrochemical DNA biosensor for the detection of Mycobacterium tuberculosis. J. Mater. Chem. B.

